# Consequences of inadvertent intravitreal Mitomycin C injection

**DOI:** 10.1186/s40942-018-0110-6

**Published:** 2018-02-12

**Authors:** Ahmad Mirshahi, Alireza Lashay, Mohammad Reza Mehrabi Bahar, Mojtaba Abrishami

**Affiliations:** 1 Eye Research Center, Farabi Eye Hospital, Tehran University of Medical Sciences, Tehran, Iran; 2 Eye Research Center, Khatam Eye Hospital, Mashhad University of Medical Sciences, Qarani Blvd., Mashhad, 9195965919 Iran

**Keywords:** Mitomycin C, Retinal toxicity, Intravitreal injection, Electroretinography

## Abstract

**Background:**

Mitomycin C (MMC) as an alkylating agent is miscellaneous an antineoplastic, antibiotic and ophthalmic agent. Here we aim to report a case of inadvertent intravitreal MMC injection instead of Avastin in case of diabetic macular edema.

**Case presentation:**

A 53 years old woman was planned to receive intravitreal Avastin injection, but accidentally, 0.05 ml of MMC 0.2% was injected. Best corrected visual acuity (BCVA) was 20/160 before injection. After 2 days, patient was referred to a tertiary referral eye center. BCVA was hand motion at presentation. Intraocular pressure was 4 mmHg. In slit lamp exams, conjunctival injection, corneal edema, Descemet fold, anterior chamber and anterior vitreous cells were presented. Pars plana vitrectomy with peripheral vitreous shaving and silicone oil tamponade was performed. Electroretinography showed undetectable responses. Ultrasound biomicroscopy showed ciliary body shortening and detachment. Optical coherent tomography showed diffuse retinal edema the day after surgery, subretinal fluid pockets in 2 weeks, and atrophy with undetectable and intertwined layers 2 months later. Gradually, like the retina, iris became atrophic and pigments were dispersed diffusely over the lens and endothelium.

**Conclusion:**

MMC is showed to be severely toxic to intraocular tissues. In our case, iris and ciliary body became atrophic. Ciliary body detachment induced hypotony. Moreover, MMC induces retinal necrosis and atrophy. Visual outcome is profoundly poor.

## Background

Mitomycin C (MMC) is miscellaneous an antineoplastic, antibiotic and ophthalmic agent. It acts as an alkylating agent and produces DNA cross-linking, mostly on guanine and cytosine pairs, inhibiting synthesis of DNA and RNA and degrading DNA by nuclear lysis and formation of giant cells. MMC has its maximum effect against cells in late G and early S phases [1]. In glaucoma filtration surgeries, MMC applied topically, and is believed to alter conjunctival vascular endothelium and inhibit fibroblast proliferation [2]. Systemic absorption following ocular administration is unknown, however, it should be far lower than when it is administered parenterally as a chemotherapy agent [1]. The use of MMC in keratorefractive surgeries became widespread as it effectively reduced haze formation after surgery and improve the predictability of visual outcomes following refractive surgery [3]. Moreover, in glaucoma and pterygium surgeries, topical solution is applied by sponges to surgical site, usually for 2 min [2].

Lenticular changes and cataract is reported in topical applications [2]. Inadvertent corneal and/or scleral damage, including thinning or perforation, may occur with use of MMC solution in concentrations > 0.2 mg/mL or for time periods > 2 min [2]. There are several reports of complications after MMC usage even many years after topical application of the drug [4, 5]. As a result, many studies were designed to evaluate the effect of MMC usage in refractive surgery on the corneal endothelium. Some studies have reported significant corneal endothelial toxicity, while others failed to detect significant change in corneal endothelial density or morphology [6].

Previous reports were focused on topical application of the MMC. Here we report accidental intravitreal MMC injection in a case of diabetic macular edema instead of Bevacizumab.

## Case description

A 53 years old woman with diabetic macular edema in her right eye and decreased vision to 20/160 was recommended to have an intravitreal bevacizumab injection. In the operating theater, however, 0.05 ml of 0.2% Mitomycin C solution inadvertently was injected intravitreally. The ophthalmologist immediately referred the patient to the emergency department of a tertiary referral center for urgent vitrectomy, but the patient presented to the hospital 2 days later. Due to the distance, she came late till visual loss became severe.

At presentation, visual acuity was hand motion (HM), with relative afferent pupillary defect (RAPD). In slit lamp exam, conjunctival injection, corneal edema with Descemet fold, and deep anterior chamber with inflammatory cell was seen. Intraocular pressure (IOP) was 4 mmHg. Cataract was nuclear sclerosis, and red reflex was reduced. Anterior vitreous cell was observed. Optic disc was round in shape, erythematosus, with sharp borders and cup/disc ratio of 0.4. Fundus exam revealed macular edema with hard exudates and diabetic retinopathy as mild to moderate non proliferative diabetic retinopathy. Visual acuity of her left eye was 20/20 with mild non proliferative diabetic retinopathy.

23 gauge pars plana deep vitrectomy with posterior vitreous detachment induction and peripheral vitreous shaving was performed. Retinal venular tortuosity, arterial narrowing and diffuse retinal edema were seen. No retinitis or infarction was observed. As the patient was hypotonic, silicone oil tamponade was used. Sclerotomies were sutured and subtenon triamcinolone acetonide (TriamHEXAl, Hexal AG, Holzkirchen, Germany) was injected.

**Fig. 1:**
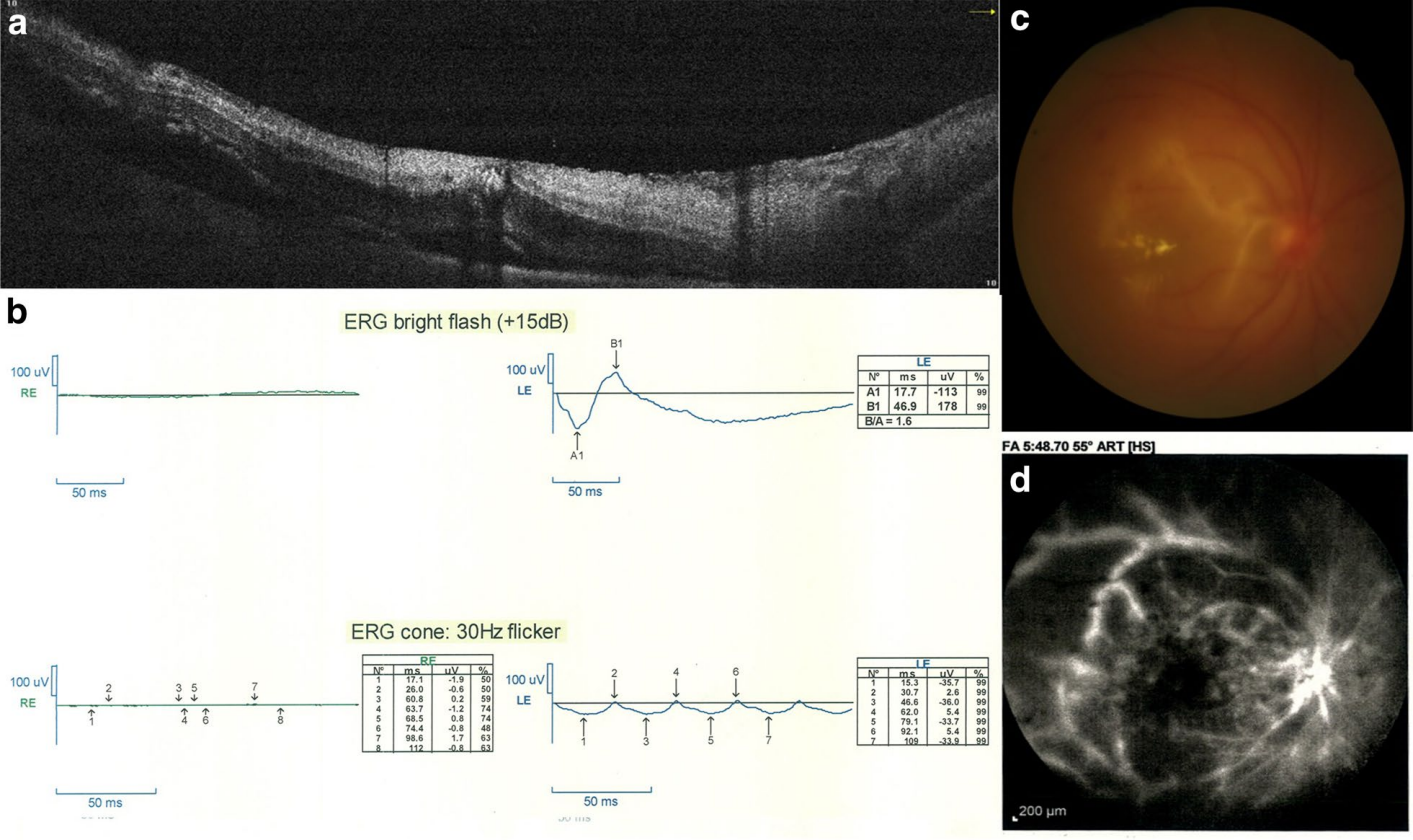
**a** Optical coherent tomography image of the macula, showing retinal thickening, cystoid intraretinal spaces, and small subretinal fluid pockets. **b** Electroretinogram of the right eye shows both dark adopted maximal combined response and light adopted 30 Hz flicker response shows undetectable electroretinogram response. **c** Fundus photograph showing macular exudates, dilated veins and retinal hemorrhages. **d** Fluorescein angiography shows vascular staining and leakage and hypofluorescence due to ischemia

**Fig. 2:**
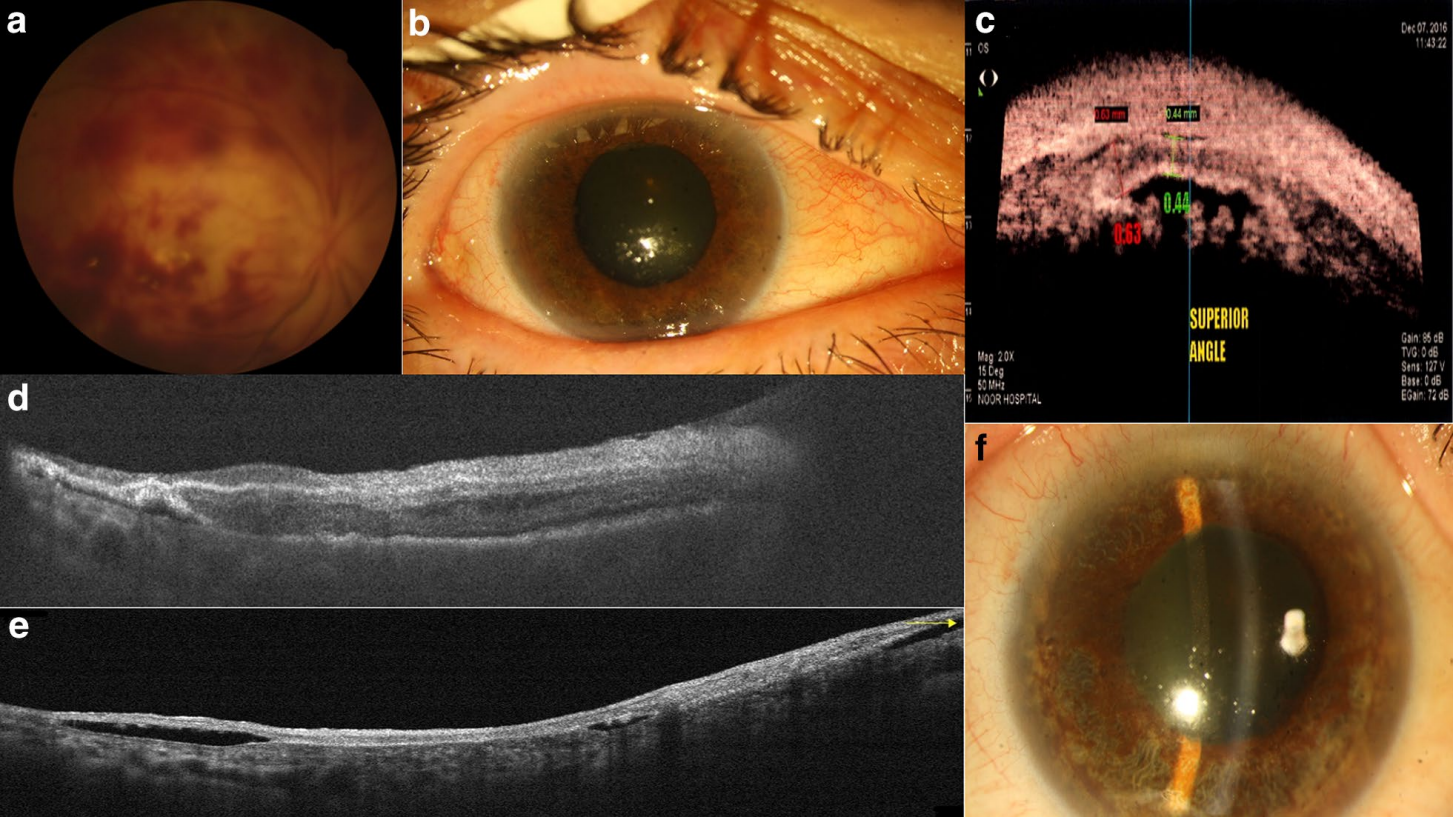
**a** Fundus photograph showing patchy intraretinal hemorrhages 20 days after vitrectomy. **b** Slit photograph showing mild corneal edema and mild patchy iris atrophy. **c** Ciliary body detachment and cleft and atrophy in UBM scan. **d** OCT scan shows small pockets of subretinal fluid. Retina showed atrophic changes in temporal retina and also reduce in retinal thickness. Edema was reduced 22 days after MMC injection. **e** Two months later, retina became atrophic and all layers became intertwined. Subretinal fluid was remained. **f** Iris atrophy progressed and pigment dispersed all over in the anterior chamber over the lens, iris and endothelium

**Fig. 3:**
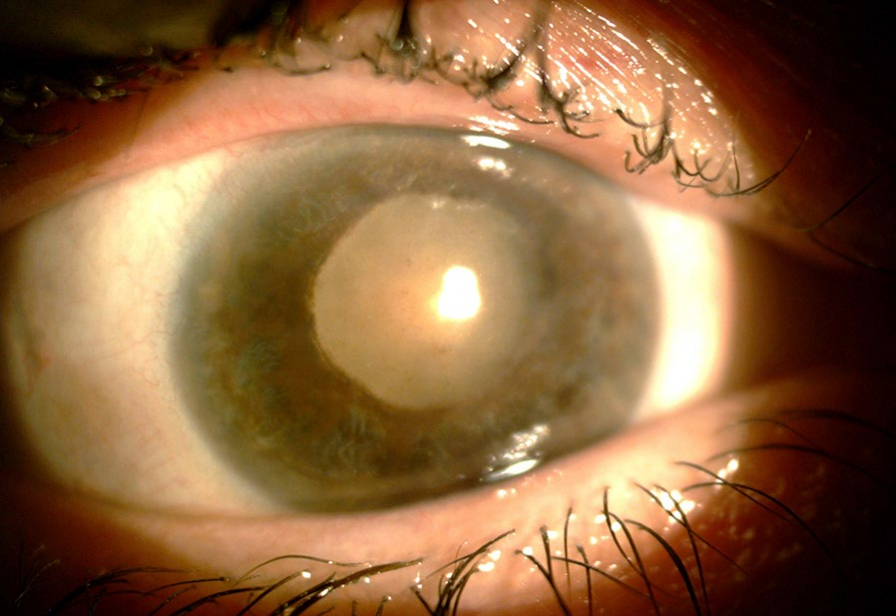
Slit lamp photograph of the patient showing mature cataract, mild corneal edema and diffuse spongy iris atrophy, one year after intravitreal MMC injection

The day after vitrectomy, visual acuity was HM. Cornea was still edematous. Retina was attached and featureless, and arteries became narrower. IOP was 8 mmHg. Fundus photograph, fluorescein angiography, optical coherent tomography (OCT) and electroretinography were requested (Fig. 1). In fluorescein angiography, due to media opacity, details were not evaluated. Only large vessels were visible. Vascular stating and tortuosity was remarkable. In OCT, although the quality of the images was low due to haziness of the media, retina was shown edematous with cystic changes. In electrophysiology, undetectable flat ERG response was recorded in right eye and normal responses in the left eye. Patient was discharged and followed by 10 days topical Ciprofloxacine (Ciplex, Sina Darou, Tehran, Iran) every 8 h, high frequency (every 3 h) topical Betamethsone (Betasonate, Sina Darou, Tehran, Iran) and every 8 h cycloplegic as Homatropine (Homydrin, Sina Darou, Tehran, Iran). Moreover, topical NaCl 5% (Natrisalt, Sina Darou, Tehran, Iran) eye drop every 6 h and ointment for nights was prescribed. Two weeks later, IOP reduced to 6 mmHg. The ultrasound biomicroscopy (UBM) showed ciliary body shortening and detachment. Retina was attached with patchy intraretinal hemorrhages. In OCT evaluation, edema was less and small pockets of subretinal fluid were seen. In temporal to macula, retinal thickness was reduced and atrophy was initiated (Fig. 2a–d) After 2 months, visual acuity declined to poor light perception. Corneal edema was mild. Iris became atrophic with pigments distributed on the endothelium, over the lens and also the spongy iris stroma. Retina was clinically attached and became atrophic and featureless. Optic disc became semi-pale. In OCT, atrophy with all layers became intertwined and indistinguishable (Fig. 2e–f). After 1 year, visual acuity was no light perception. IOP was 4 mmHg. Patient had mature cataract, diffuse iris atrophy, mid dilated iris and mild corneal edema (Fig. 3). In specular microscopy, cell density was near half of the other eye (1670 vs. 2409) and mean cell size was half larger (598 vs. 415 µm^2^).

## Discussion and conclusions

Mitomycin C is mainly as an alkylating agent. MMC has a variety of specific biological effects in mammalian cells, including selective inhibition of DNA synthesis, mutagenesis, and stimulation of genetic recombination, chromosome breakage. Although DNA alkylation may occur at any stage in the cell cycle, it affects DNA synthesis, particularly during the late G1 and S phases [8]. MMC, therefore, works as a genotoxic antibiotic, and at high concentrations, cellular RNA and protein syntheses are also suppressed [9]. Therefore, it induces apoptosis and necrosis in high doses.

In an electroretinography and also histopathologic study, Kawashima et al. evaluate the retinal toxicity of MMC injection in the rat eye. Three different concentrations of MMC (0.2, 0.3 and 0.4 mg/ml) were injected into either the vitreous cavity or the anterior chamber of the experimental eyes. A full-field electroretinogram was recorded before injection and 2, 4 and 7 days after injection. The retinas of all eyes were examined by light and electron microscopy. They have found no evidence of electroretinography and histologic changes 2 and 7 days after injection of 0.4 mg/ml of MMC into the anterior chamber. However, profound electroretinography changes did follow injection of the drug into the vitreous. These were absent with the 0.2-mg/ml solution at 7 days, mild with the 0.3-mg/ml solution at 7 days and profound with the 0.4-mg/ml solution as early as 2 days. Intravitreal injection of 0.4 mg/ml, however, showed selective degeneration of Müller cell process at day 2, retinal pigment epithelium changes at day 4 and irregular arrangement of the outer nuclear layer and photoreceptors at day 7. It was concluded that intravitreal injection of MMC in high doses, as used as routine clinical doses, could cause retinal functional and histological disorders [7].

In a case report, Nuyts et al. claimed that there is a correlation between diffusion of MMC into the vitreous cavity and retinal vasculature occlusion. In a 45-year-old male who underwent a trabeculectomy with MMC for a secondary glaucoma after complicated cataract surgery, due to early bleb failure, a needling procedure with subconjunctival injection of MMC was performed. Five days postoperatively, a combined occlusion of both central retinal artery and vein occlusion occurred. They have concluded that a relation between the diffusion of MMC into the vitreous cavity, because of opening of the posterior capsule, and the development of retinal toxicity may exist [10].

In this case report, we have reported the outcomes of inadvertent intravitreal MMC injection. MMC application in the ophthalmology have been evaluated mostly in the field of ocular surface and orbital surgeries. Reported side effects in this issue is limited mostly on ocular surfaces or corneal endothelium or cataract formation. The only available report was in a case who had undergone vitrectomy after injection. In that case the patient suffered cystoid macular edema, iris atrophy and mild corneal temporal edema 6 months after vitrectomy. It was a photo essay and comprehensive report of that case was not available [11]. In our patient, due to distance, patient was referred late to our center. Although we performed vitrectomy less than 4 h from the first visit, retina became necrotic and atrophic. In early phase retinal edema converted to intraretinal hemorrhages. Electroretinography was undetectable since the early phase. Gradually IOP decreased and subretinal fluid pocket were appeared. Iris became atrophic and pigments dispersed in the anterior chamber. Although we performed deep vitrectomy, used long term topical and subtenon corticosteroid, and also cycloplegic, no one could arrest the inflammatory and destructive effects of intravitreal injection of MMC. One year follow up also showed toxic effects of MMC or inflammatory responses, continued to affect the ocular tissues. Lens became cataractous and iris became atrophied. As electroretinography showed, the MMC may affect retina very soon. It also affects profoundly on uveal tissue as iris atrophy progresses and iris became atrophic.

## References

[1] Lockwood A, Brocchini S, Khaw PT (2013). New developments in the pharmacological modulation of wound healing after glaucoma filtration surgery. Curr Opin Pharmacol.

[2] Bindlish R, Condon GP, Schlosser JD, D’Antonio J, Lauer KB, Lehrer R (2002). Efficacy and safety of mitomycin-C in primary trabeculectomy: five-year follow-up. Ophthalmology.

[3] Schipper I, Suppelt C, Gebbers JO (1997). Mitomycin C reduces scar formation after excimer laser (193 nm) photorefractive keratectomy in rabbits. Eye (Lond).

[4] Wan Norliza WM, Raihan IS, Azwa JA (2006). Scleral melting 16 years after pterygium excision with topical Mitomycin C adjuvant therapy. Cont Lens Anterior Eye.

[5] Tsai YY, Lin JM, Shy JD (2002). Acute scleral thinning after pterygium excision with intraoperative mitomycin C: a case report of scleral dellen after bare sclera technique and review of the literature. Cornea.

[6] Teus MA, de Benito-Llopis L, Alió JL (2009). Mitomycin C in corneal refractive surgery. Surv Ophthalmol.

[7] Kawashima S, Mizota A, Adachi-Usami E, Kimura T. Effects of mitomycin C on the rat retina. Doc Ophthalmol. 1996–1997;92(3):229–41. 10.1007/BF02583294 9181350

[8] Verweij J, Pinedo HM (1990). Mitomycin C: mechanism of action, usefulness and limitations. Anticancer Drugs.

[9] Tomasz M (1995). Mitomycin C: small, fast and deadly (but very selective). Chem Biol.

[10] Nuyts RM, Van Diemen HA, Greve EL (1994). Occlusion of the retinal vasculature after trabeculectomy with mitomycin C. Int Ophthalmol.

[11] Ryoo NK, Kim MK, Wee WR (2013). Consequences of accidental mitomycin C intraocular injection. JAMA Ophthalmol.

